# Quantitative real-time PCR as a promising tool for the detection and quantification of leaf-associated fungal species – A proof-of-concept using *Alatospora pulchella*

**DOI:** 10.1371/journal.pone.0174634

**Published:** 2017-04-06

**Authors:** Alexander Feckler, Anne Schrimpf, Mirco Bundschuh, Felix Bärlocher, Patrick Baudy, Julien Cornut, Ralf Schulz

**Affiliations:** 1 Institute for Environmental Sciences, University of Koblenz-Landau, Landau, Germany; 2 Department of Aquatic Sciences and Assessment, Swedish University of Agricultural Sciences, Uppsala, Sweden; 3 Department of Biology, Mt. Allison University, Sackville, Canada; 4 Laboratoire Interdisciplinaire des Environnements Continentaux, UMR CNRS 7360, Université de Lorraine, Metz, France; 5 Department of Experimental Limnology, Leibniz-Institute of Freshwater Ecology and Inland Fisheries (IGB), Stechlin, Germany; 6 MARE–Marine and Environmental Sciences Centre, Department of Life Sciences, University of Coimbra, Coimbra, Portugal; Oklahoma State University, UNITED STATES

## Abstract

Traditional methods to identify aquatic hyphomycetes rely on the morphology of released conidia, which can lead to misidentifications or underestimates of species richness due to convergent morphological evolution and the presence of non-sporulating mycelia. Molecular methods allow fungal identification irrespective of the presence of conidia or their morphology. As a proof-of-concept, we established a quantitative real-time polymerase chain reaction (qPCR) assay to accurately quantify the amount of DNA as a proxy for the biomass of an aquatic hyphomycete species (*Alatospora pulchella*). Our study showed discrimination even among genetically closely-related species, with a high sensitivity and a reliable quantification down to 9.9 fg DNA (3 PCR forming units; LoD) and 155.0 fg DNA (47 PCR forming units; LoQ), respectively. The assay’s specificity was validated for environmental samples that harboured diverse microbial communities and likely contained PCR-inhibiting substances. This makes qPCR a promising tool to gain deeper insights into the ecological roles of aquatic hyphomycetes and other microorganisms.

## Introduction

Aquatic hyphomycetes are a polyphyletic group of true fungi of paramount significance in the process of leaf litter decomposition in freshwater ecosystems [1, 2]. Contaminants like fungicides and pharmaceuticals potentially threaten the functioning of this group, suggesting that aquatic hyphomycetes may be suitable bioindicators to assess anthropogenic stress [3–5]. To better understand the contribution of individual aquatic hyphomycete species to leaf litter decomposition and to estimate the impact of anthropogenic stress, knowledge of fungal community composition and of performances (e.g., biomass production) by individual species is essential, given the substantial interspecific variability among aquatic hyphomycetes [6]. Traditionally, the characterization of aquatic hyphomycete communities has been based on the morphology of asexually produced propagules (i.e., conidia). This approach, however, has several shortcomings including the reliance on a reproductive life phase [7] and analogous morphology of conidia from different species (cf. [8, 9]). Additionally, conidia only provide information on community composition and sporulation rates of individual aquatic hyphomycete species, which does not necessarily correlate with fungal biomass [10]. Total fungal biomass is usually estimated by quantifying ergosterol levels [11]. Although maximum ergosterol content may correlate with the community’s maximum sporulation rate [12], such a correlation does not exist at all stages of fungal succession or when addressing individual aquatic hyphomycete species [10, 13].

Molecular techniques, which have been suggested as indispensable tools to investigate the evolution and ecology of aquatic hyphomycetes [14], may help circumvent these shortcomings: since nucleic acids can be characterized during all stages of the fungal life cycle and thus also during non-sporulating phases, species richness could possibly be determined more exhaustively. In this context, quantitative real-time polymerase chain reaction (qPCR) shows promise, allowing both identification and quantification of individual species by correlation with hyphal biomass and ITS copies (cf. [15]). Applying qPCR assays to studies of leaf litter decomposition is not entirely new (e.g., [6, 16]). However, previously applied methods were based on SYBR Green as fluorescence dye binding to the double-stranded DNA. This technique is not highly selective, since SYBR Green not only binds to the PCR product, but also to primer dimers formed during PCR reactions. Additionally, it seems to lack consistently reproducible quantification when the target gene is expressed at low levels [17]. These shortcomings in specificity as well as sensitivity limit the informative value when compared to highly specific TaqMan probes [16–19].

The present study, therefore, reports the development of a species-specific TaqMan MGB qPCR method allowing accurate quantification of the DNA (used as a proxy for biomass) of the aquatic hyphomycete *Alatospora pulchella*. This species is found globally on decomposing leaf litter in streams and therefore of relevance for the decomposition process [20–22], and it belongs to a genus preferentially fed on by leaf-shredding invertebrates [23, 24]. The protocol was validated against 24 aquatic hyphomycete species tested both individually and in mixtures. In addition, the protocol was tested in the presence of DNA extracts of leaf material from a previous study that contained up to 14 aquatic hyphomycete species [24] to validate the specificity of the assay in environmental samples.

## Materials and methods

### Genetic classification of aquatic hyphomycete strains

To ensure the exclusive use of pure strains of aquatic hyphomycetes during the present study, the ribosomal internal transcribed spacer (ITS) region [25] was sequenced for each strain. The method is fully described in S1 File.

### Design of qPCR primers and probe

The ITS sequences of pure strains (Table 1) were aligned using the ClustalW algorithm [26] and sequence motifs suitable for a species-specific analysis of *A*. *pulchella* were identified applying the software Primer express 2.0 (Applied Biosystems, Foster City, California). Two primer binding-sites and one probe binding-site in the ITS-1 region were further tested for species-specificity: forward primer AlaPul-ITSfor (5’-TTGGAGGTCCGGCTGTGT-3’), reverse primer AlaPul-ITSrev (5’-TCCGAGGTCAACCTTAAAAAAATT-3’) and TaqMan probe AlaPul-ITSprobe (5’-CCTGCCAGCAACC-3’). The probe was labeled with 6-carboxy-fluorescein at the 5’-end and MGBNFQ at the 3’-end as fluorescent reporter dye and non-fluorescent quencher, respectively.

**Table 1:** Information on aquatic hyphomycete strains.

Species identified		Strain number	Provenance	Inclusivity (I) /	Accession numbers	Ct value
morphologically	genetically (previously published GenBank accession numbers)			exclusivity (E) panel	of strains in present study	
*Alatospora acuminata*	*Alatospora acuminata* (AY204588)	180–1658	France	E	KU519109	-
*Alatospora acuminata*	*Alatospora acuminata* (AY204588)	CCM F-12186^a^	Slovakia	E	AY204588	-
*Alatospora acuminata*	*Alatospora acuminata* (AY204588)	DSM 104360^b^	Germany	E	-	-
*Alatospora flagellata*	*Alatospora flagellata* (KC834041)	182–1679	France	E	KU519110	-
*Alatospora acuminata*	*Alatospora pulchella* (KF730799)	130–1663	France	I	KU519111	12.88
*Alatospora pulchella*	*Alatospora pulchella* (KU519111)	DSM 103606^b^		I	KY616856	31.09
*Anguillospora crassa*	*Anguillospora crassa* (AY204581)	130–1659	France	E	KU519112	-
*Anguillospora crassa*	-	DSM 104363^b^	Germany	E	-	
*Anguillospora filiformis*	*Anguillospora filiformis* (JX089461)	CCM F-19787^a^	Canada	E	KU519113	-
*Articulospora atra*	*Articulospora atra* (KP234353)	CCM F-0684^a^	Czech Republic	E	KU519114	-
*Clavariopsis aquatica*	-	DSM 104362^b^	Germany	E	-	
*Clavatospora longibrachiata*	-	DSM 104364^b^	Germany	E	-	
*Clavatospora longibrachiata*	-	DSM 104365^b^	Germany	E	-	
*Heliscella stellata*	-	DSM 104357^b^	Germany	E	-	
*Lemonniera aquatica*	-	DSM 104378^b^	Germany	E	-	
*Lemonniera cornuta*	-	185–1685	France	E	KU519115	-
*Lemonniera terrestris*	-	183–1669	France	E	KU519116	-
*Neonectria lugdunensis*	-	DSM 104361^b^	Germany	E	-	
*Tetracladium breve*	*Tetracladium breve* (FJ000364)	CCM F-12505^a^	Portugal	E	KU519117	-
*Tetracladium furcatum*	*Tetracladium furcatum* (KC180668)	CCM F-11883^a^	Czech Republic	E	KU519118	-
*Tetracladium marchalianum*	-	DSM 104373^b^	Germany	E	-	
*Tetracladium maxilliforme*	*Tetracladium maxilliforme* (HM036615)	CCM F-529^a^	Czech Republic	E	KU519119	-
*Tetracladium setigerum*	*Tetracladium setigerum* (FJ000374)	CCM F-20987^a^	Canada	E	KU519120	-
*Tricladium angulatum*	*Tricladium angulatum* (AY204610)	4–1683	France	E	KU519121	-
*Tricladium chaetocladium*	*Tricladium chaetocladium* (KC834067)	180–1646	France	E	KU519122	-
*Tricladium splendens*	*Tricladium splendens* (FJ000400)	TRSL162-1436^c^	USA	E	KU519123	-
*Tricladium splendens*	-	DSM 104369^b^	Germany	E	-	
*Tumularia aquatica*	-	DSM 104371^b^	Germany	E	-	
*Tumularia tuberculata*	-	DSM 104368^b^	Germany	E	-	

Aquatic hyphomycete strains (maintained as pure cultures; identified morphologically and genetically) used as a source of DNA. Previously published GenBank accession numbers of reference sequences used for the genetic identification of the aquatic hyphomycete strains are displayed if accessible. Furthermore, the allocation of strains to the inclusivity panel (should be detected) and exclusivity panel (should not be detected) is shown. Newly generated GenBank accession numbers of the strains used during the present study and Ct values of the qPCR are given in the last two columns. Dashes are shown where no new GenBank accession numbers were generated, genetic identification was not possible or no positive signal was detected during qPCR.

^a^Czech Culture Collection

^b^Leibniz-Institut DSMZ–Deutsche Sammlung von Mikroorganismen und Zellkulturen GmbH

^c^EcoLab—Laboratoire écologie fonctionnelle et environnement (Eric Chauvet)

### qPCR procedure

qPCR reactions were performed for all DNA extracts of the inclusivity/exclusivity panel for the established assay (Table 1) and run on a Mastercycler^®^ ep *realplex* S (Eppendorf, Hamburg, Germany). Each qPCR reaction was performed in a total volume of 25 μL, containing 5 μL template DNA, 8.7 μL sterile ddH_2_O, 1.3 μL primer-probe-mix (primers: 18 μM and probe: 5 μM in the stock), and 10 μL TaqMan Environmental Master Mix (Applied Biosystems, Foster City, California) avoiding PCR inhibition [27]. Eppendorf real-time PCR tube strips covered with Masterclear^®^ cap strips (Eppendorf, Hamburg, Germany) were used as reaction vessels for amplification and detection. The qPCR temperature profile entailed the following steps: initial denaturation (2 min at 50°C) for optimal Uracil-N-Glycosylase enzyme activity and successive activation of the DNA polymerase (10 min at 95°C), followed by 50 cycles of 15 sec at 95°C and 1 min at 60°C. Two negative controls (environmental control and extraction blank control) were included at least once in qPCR procedures to exclude any contamination.

### Sensitivity analysis and validation

As a measure of sensitivity and the quantitative range of the developed qPCR procedure, the limit of detection (LoD) and limit of quantification (LoQ) were determined by slightly modifying the method by Vrålstad et al. [28]: in total, 14 standards were prepared by a four-fold serial dilution (4^−1^, 4^−2^, …, 4^−14^) of the *A*. *pulchella* extract (measured initial concentration of 3.6 ng DNA/μL; NanoDrop 1000, NanoDrop products, Wilmington, Delaware; Table 2). For each standard, seven independent qPCR runs were conducted, following the procedure described above. To further validate the specificity of the established assay for environmental samples (i.e., leaf-associated microbial communities), leaf material from a previous study was used in which up to 14 aquatic hyphomycete species were morphologically identified [24]. The full method description is reported in S2 File.

**Table 2:** Information on *A*. *pulchella* standards, their Ct values and detection frequency.

Standard / dilution	DNA in standard (ng/μL)	DNA in PCR (ng)^a^	Estimated PFU per PCR	Mean Ct value (± SD)	Detection frequency (%) during seven independent qPCR runs
1 / 4^−1^	9.00 × 10^−1^	4.50	1.3 × 4^10^	15.20 (± 0.39)	100
2 / 4^−2^	2.25 × 10^−1^	1.13	1.3 × 4^9^	17.48 (± 0.34)	100
3 / 4^−3^	5.63 × 10^−2^	2.81 × 10^−1^	1.3 × 4^8^	19.61 (± 0.33)	100
4 / 4^−4^	1.41 × 10^−2^	7.03 × 10^−2^	1.3 × 4^7^	21.85 (± 0.48)	100
5 / 4^−5^	3.52 × 10^−3^	1.78 × 10^−2^	1.3 × 4^6^	23.85 (± 0.23)	100
6 / 4^−6^	8.79 × 10^−4^	4.39 × 10^−3^	1.3 × 4^5^	25.89 (± 0.18)	100
7 / 4^−7^	2.20 × 10^−4^	1.10 × 10^−3^	1.3 × 4^4^	27.94 (± 0.37)	100
8 / 4^−8^	5.49 × 10^−5^	2.75 × 10^−4^	1.3 × 4^3^	30.59 (± 0.52)	100
9 / 4^−9^	1.37 × 10^−5^	6.87 × 10^−5^	1.3 × 4^2^	32.30 (± 0.79)	100
10 / 4^−10^	3.43 × 10^−6^	1.72 × 10^−5^	1.3 × 4^1^	34.59 (± 0.60)	100
11 / 4^−11^	8.58 × 10^−7^	4.29 × 10^−6^	1.3	36.87 (± 1.00)	71
12 / 4^−12^	2.15 × 10^−7^	1.07 × 10^−6^	1.3 × 4^−1^	39.25 (± 0.13)	43
13 / 4^−13^	5.36 × 10^−8^	2.68 × 10^−7^	-	-	-
14 / 4^−14^	1.34 × 10^−8^	6.71 × 10^−8^	-	-	-
Negative control	-	-	-	-	-

Dilution steps of *A*. *pulchella* extract with information on the amount of template DNA in standards as well as PCR runs, estimated PCR forming units (PFU) per PCR, Ct value (mean ± SD), and detection frequency during seven independent qPCR runs. Dashes: variables could not be quantified.

^a^ 5 μL template (per reaction) multiplied by the assigned concentration per μl

### Data analysis

Since some target DNA copies serving as templates for a PCR may be damaged or fragmented (e.g., during extraction and freeze-thaw during handling), DNA mass measurements may overestimate copy numbers [29]. Hence, the term “PCR forming units” (PFU) was introduced to clearly distinguish between copies being truly amplified during PCR and crude copy number estimates based on the measured DNA amount [30]. Based on this framework, the number of PFU per standard was calculated following the SIMQUANT assay [29]. SIMQUANT assumes a ratio of positive:negative PCR reactions of 7:3 (70% detection) during a series of PCR reactions with approximately 1 PFU. Accordingly, the standard containing 1 PFU was roughly identified and the DNA contents of the remaining standards were successively converted into respective PFU. These values were used to generate a calibration curve, where the mean Ct value for each standard (*n* = 7) was plotted as a function of the corresponding PFU. Subsequently, the LoD and LoQ were derived following the descriptions in Vrålstad et al. [28]. For a full description of the calculations for the LoD and LoQ, see S3 File. The software Real Plex 2.2 (Eppendorf, Hamburg, Germany) and R for Mac version 3.0.2 [31] were used for data analysis and preparation of figures.

## Results and discussion

### Specificity and sensitivity of the qPCR assay

The molecular characterization of the fungal strains used during the present study revealed their genetic purity. However, there were some discrepancies between molecular characterization and morphological identification of the conidia (Table 1). This may be due to similar environmental pressures resulting in convergent conidial morphology reached by different conidiogenous processes in non-related species [8]. Additionally, there is growing concern about the taxonomic reliability of fungal DNA sequences in public sequence repositories (e.g., [32, 33]) with an estimated 20% of the entries being incorrectly identified at the species level [34].

Nevertheless, the theoretical specificity analysis of the assay, based on the Basic Local Alignment Search Tool (BLASTn)-algorithm at the National Center for Biotechnology Information (NCBI), resulted in a ≥ 99% query cover with the target species (e.g., GenBank Accession no. KF730806, E-value: 0.0, Identity: 100%). More importantly, sequences for species displaying the highest genetic similarity to *A*. *pulchella* showed ≥ 2 base mismatches at the target site for the probe (for description, see S4 File and S5 File; Figures A and B in S4 File). The only exception was found in *Flagellospora curvula* (GenBank Accession no. AY729939), which did not show any mismatch with the probe’s sequence (Figure B in S4 File). A BLASTn-search conducted with the respective sequence, however, revealed a higher similarity to *Alatospora* sequences than to any other of the genus *Flagellospora*. Accordingly, a direct comparison of the doubtful *F*. *curvula* sequence with additional deposited sequences of the same species showed a high degree of mismatch, including the primer and probe regions (results not shown). Hence, it is likely that the *F*. *curvula* sequence was deposited under the wrong species name. Morphological misidentifications seem rather unlikely given the generally marked differences in size and shape of *Alatospora* and *Flagellospora* conidia [35, 36]. Also, publicly available sequences from morphologically-related species of the genus *Alatospora*, namely *A*. *acuminata*, *A*. *constricta*, and *A*. *flagellata*, displayed ≥ 2 base mismatches for the 12 base pair long probe sequence motif (Figure B in S4 File). In contrast, *A*. *pulchella* sequences showed no base substitution for the same motif. Therefore, false-positive amplifications for species of the exclusivity panel are highly unlikely given the high sensitivity of MGB probes towards mismatches at the annealing site [37, 38]. Additionally, mismatches were apparent for the forward primer-template pairs of species of the exclusivity panel (≥ 2 mismatches; Figure B in S4 File), further reducing the possibility of false-positive amplifications.

**Fig 1:**
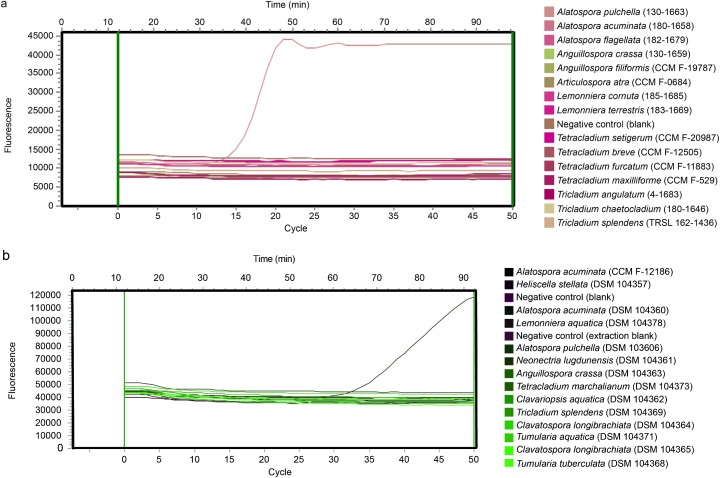
**1a and 1b. Fluorescence profiles for the 6-carboxy-fluorescein fluorophore (FAM) as a function of PCR cycle and time.** Each tested species and the negative controls are represented by a different color. A signal was classified as true positive if it was substantially higher than the initial fluorescence baseline [39], which was set automatically and individually for each sample by the qPCR device. Please note that the higher Ct value of *A*. *pulchella* in Fig 1b resulted from an extract with less template DNA.

In agreement with this theoretical specificity, our results demonstrated that the species-specific primers and TaqMan probe fulfilled all requirements for the qPCR application as they exclusively amplified the target DNA for the strains of the inclusivity panel and this at a Ct value as low as 12.88 (Fig 1A and 1B; Table 1). None of the species included in the exclusivity panel showed a positive signal during the qPCR runs (Table 1). The empirical verification of the assay’s specificity should ideally have covered a broader spectrum than 24 aquatic hyphomycete species. Nonetheless, as species with a close genetic relationship to *A*. *pulchella–*namely *A*. *acuminata*, and *A*. *flagellata* (Table A in S5 File)–did not show any positive signal during the qPCR runs, the developed method is reasonably specific at the species level.

**Fig 2:**
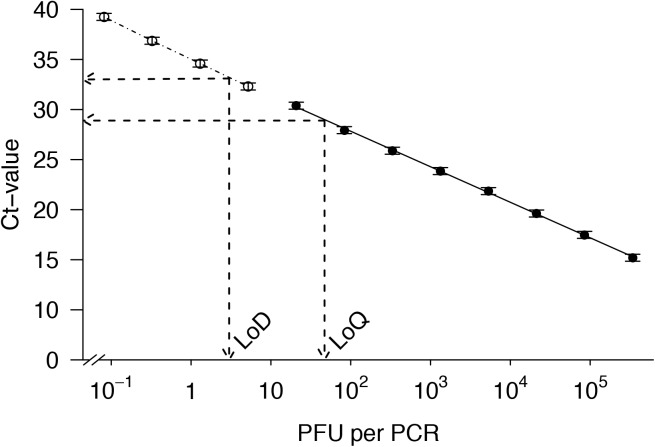
Standard curve for quantitative *A*. *pulchella* standards. The standard curve (solid line) is displayed as mean Ct values (± SD; *n* = 7) of quantitative standards of *A*. *pulchella* (dilutions 4^−1^ to 4^−8^; filled circles) as a function of the calculated number of PCR forming units (PFU) in each PCR reaction (linear model *r*^*2*^ was 0.9994; *p* < 0.001). Non-quantitative calibrants (dilutions 4^−9^ to 4^−12^; open circles) that are not included in the standard curve are also visualized and connected by a dotted line. The limit of detection (LoD; 3 PFU; 9.9 fg DNA) and limit of quantification (LoQ; 47 PFU; 155.0 fg DNA) are indicated by arrows.

The method showed a high sensitivity for the target species. Calculations resulted in a LoD of 3 PFU corresponding to reliable detection down to 9.9 fg DNA. The quantitative range of the method covered the first eight standards (Fig 2; S3 File), where each standard differed by approximately two Ct units from the previous one indicating a four-fold difference in copy numbers as shown in Table 2 (cf. [40]). This demonstrates reliable quantification of DNA down to 47 PFU (= LoQ; 155.0 fg DNA; S3 File); the method is therefore feasible even with low target copy numbers. The quantitative measurements suggest a high reliability of the qPCR method with a high coefficient of determination (*r*^2^ = 0.9994), low standard deviations of Ct values as well as repeatedly detected true-positive signals for the diluted extract down to a factor of 4^−8^ (Fig 2; Table 2). Hence, concerns by Vrålstad et al. [28] that such quantitative assessments strongly depend on the accuracy of the standard curve, the degree of measurement uncertainty and the precision of added standard volumes were of minor importance during the present study but should be taken into account during routine applications.

Finally, the assay also showed its high specificity in environmental samples (S2 File) containing diverse communities of aquatic hyphomycetes and bacteria [24] and likely including PCR inhibitors such as humic substances leaching from leaf litter [27, 41]. The leaf-associated microbial communities of two out of the three tested environmental samples (i.e., C2 and C3) did not contain the target species *A*. *pulchella*, since the Ct values of ≥38 indicated these peaks were below the assay’s LoD and thus false-positives. The third sample (C1), on the other hand, contained a certain amount of target copies, revealed by a Ct value of 25.7 indicating 9.4–150 fg template DNA of *A*. *pulchella* (Table A in S2 File). This pattern was somewhat unexpected, since the morphological identification of the aquatic hyphomycete community associated with the leaf material only revealed the presence of *A*. *acuminata* and not of *A*. *pulchella* [24]. This discrepancy may be due to misidentification, attributable to the high number of *Alatospora* conidia in the samples [24] and the similarity of the conidial morphologies of *A*. *acuminata* and *A*. *pulchella* [42]. Nevertheless, the assay demonstrated its specificity for *A*. *pulchella* by showing true-positive results when the target species was present and each standard differed on average by approximately four Ct values from the previous one indicating a 16-fold difference in copy numbers [40]. This is in accordance with the added amount of template DNA (Table A in S2 File).

### Applicability of the qPCR assay in leaf litter decomposition research

As highlighted earlier, molecular approaches overcome several shortcomings of traditional methods in leaf litter decomposition research. Our qPCR assay for *A*. *pulchella* is a proof-of-concept for the suitability of this technique. When additional species-specific protocols become available, this approach will allow a deeper understanding regarding the role of individual aquatic hyphomycete species in the leaf litter decomposition process. When linking aquatic hyphomycete diversity and ecosystem functioning, most previous studies have been limited in their ability to track performances of individual species within complex microbial assemblages (cf. [6]): simply knowing changing species numbers in the aquatic hyphomycete community and the overall ergosterol content does not necessarily explain shifts in microbial-mediated leaf litter decomposition (e.g., [43]). It is essential to assess the biomasses of individual species to uncover their contributions to ecosystem processes [44]. qPCR methods might bridge this gap, as the amount of DNA (representative for fungal biomass) for each species can be determined accurately. This procedure could allow estimating the contributions of individual species to total community performance and thereby lead to a better understanding of the potential linkages between fungal diversity and ecological functions during leaf litter decomposition. This approach is not limited to aquatic hyphomycetes but can be extended to other microbial groups typically ignored by traditional approaches but contributing to leaf litter decomposition (e.g., Chytridiomycetes, Oomycota and other fungus-like organisms [16]).

Furthermore, qPCR might result in a better mechanistic understanding of trophic coupling at the base of heterotrophic food webs. As shown, for instance, by Arsuffi and Suberkropp [23], shredders distinguish between fungal species on leaf litter, potentially triggered by the production of feeding stimulants or distasteful compounds. This concept was recently applied to explain fungicide-induced shifts in microorganism-mediated leaf palatability for *Gammarus fossarum* [3, 24], a key shredder in low-order streams of Europe [45]. The study of Zubrod et al. [3], however, was based on spore counts, which does not necessarily correlate with the biomass of the respective aquatic hyphomycete species [10]. Applying qPCR could result in deeper insights into shredder food preferences by estimates of single-species biomasses linked to prior knowledge of preferred species.

## Conclusions

The presented TaqMan qPCR assay showed its species-specificity and sensitivity–even for closely related species no false-positive results were observed. Most shortcomings associated with qPCR are controllable and/or avoidable, and the validity of the assay was shown for environmental samples. Thus, qPCR is a promising tool for a species-specific identification and quantification of microorganisms involved in the decomposition of leaf litter. However, the method’s applicability will have to be further investigated by cross-validation based on an even broader spectrum of microorganisms.

## Supporting information

S1 File Description of DNA extraction, amplification, and sequencing. (DOCX)

S2 File Description of the assays’ validation for environmental samples (DOCX)

S3 File Calculation of the limit of detection and limit of quantification. (DOCX)

S4 File Theoretical validation of the qPCR assay’s specificity. (DOCX)

S5 File Estimates of evolutionary divergences among sequences. (DOCX)
