# Supplementary material for: Quantitative real-time PCR as a promising tool for the detection and quantification of leaf-associated fungal species – A proof-of-concept using Alatospora pulchella
Source: PLoS One. 2017 Apr 6;12(4):e0174634. doi: 10.1371/journal.pone.0174634 (PMC5383034; doi:10.1371/journal.pone.0174634)
Supplement: S1 File — (DOCX) [file pone.0174634.s001.docx]

**S1.** DNA extraction, amplification, and sequencing

Aquatic hyphomycete strains, used as a source for live spores during the present study (Table 1), were maintained on 2% malt extract agar (VWR, Germany) at 16 ± 1 °C for 2-3 weeks. DNA was extracted from living mycelium generally following the method detailed in Vrålstad et al. [1]: Mycelium from an approximately 5 × 5 mm^2^ area was scraped off at the growing edge of the colony on the malt agar using a sterile scalpel, transferred into 700 μL CTAB buffer (20 g CTAB per litre, 1.4 M NaCl, 0.1 M Tris-HCl, 20 mM Na_2_EDTA) and homogenized using a TissueLyser II (Qiagen, Hilden, Germany). After homogenization, samples were frozen at -80 °C for 15 min, followed by thawing at 65 °C. Subsequently, 10 μL RNAse solution (10 mg/mL) were added and samples were mixed. After incubation at 37 °C for 30 min on a thermal shaker, 10 μL proteinase K (10 mg/mL) were added and samples were mixed and incubated at 55 °C for 30 min. Tubes were then vortexed and centrifuged for 5 min at 12,000 × *g*. Approximately 600 μL of the supernatant were transferred to fresh tubes, an equivalent volume of chloroform was added and samples were vortexed. Tubes were centrifuged for 15 min at 18,000 × *g*, 400 μL of the supernatant were transferred to fresh tubes and 300 μL of ice-cold isopropanol were added. After mixing by careful inversion, samples were incubated for 20 min at room temperature and subsequently centrifuged for 10 min at 18,000 × *g* to pellet the DNA. The supernatant was carefully discarded, and the pellet was cleaned in 300 μL of ice-cold ethanol (70%) by centrifugation for 5 min at 18,000 × *g*. Again, the supernatant was discarded carefully and pellets were dried for 15 min at 50 °C. Finally, the pellets were re-suspended in 50 μL sterilized ddH_2_O. The amount of DNA in the extracts was measured using NanoDrop 1000 (NanoDrop products, Wilmington, Delaware). As recommended [cf., 1], potential carry-over contamination was considered by two negative controls (i.e., environmental control and extraction blank control). Both types of controls were included at least once in further qPCR operational procedures to exclude any contamination.

To verify species identity of the DNA-extracts, a sequence analysis was performed. As recommended for fungi [cf., 2], the ribosomal (ITS) region was chosen for amplification and sequencing using the primer pair ITS1/ITS4 [3]. The PCR was performed in a total volume of 25 μL containing 0.625 U GoTaq DNA Polymerase, 1X Colorless GoTaq^®^ Flexi Buffer, 2 mM MgCl_2_ (all Promega, Mannheim, Germany), 0.24 mM of each dNTP (Fermentas, St. Leon-Rot, Germany), 0.2 μM of each primer and 5 μL of DNA extract. The PCR temperature profile consisted of the following steps: initial denaturation (2 min at 94 °C); 38 cycles of 45 sec at 94 °C, 45 sec at 55 °C, and 90 sec at 73 °C; followed by a final elongation (10 min at 70 °C). PCR amplicons were commercially sequenced by SeqIT (Kaiserslautern, Germany) on a 3730 DNA Analyzer eight capillary sequencer (Applied Biosystems, Foster City, California) in both directions. Obtained sequences were manually proofread using Geneious Basic 7.1.6 [4] and subjected to the Basic Local Alignment Search Tool (BLASTn)-algorithm at the National Center for Biotechnology Information (NCBI) for similarity analysis and species identification. Sequences for pure aquatic hyphomycete cultures were uploaded to the NCBI database (Table 1).

References cited in S1

1. Vrålstad T, Knutsen AK, Tengs T, Holst-Jensen A. A quantitative TaqMan^®^ MGB real-time polymerase chain reaction based assay for detection of the causative agent of crayfish plague *Aphanomyces astaci*. Vet. Microbiol. 2009; 137:146-55.

2. Schoch CL, Seifert KA, Huhndorf S, Robert V, Spouge JL, Levesque CA, et al. Nuclear ribosomal internal transcribed spacer (ITS) region as a universal DNA barcode marker for fungi. P. Natl. Acad. Sci. 2012; 109:6241-6.

3. White TJ, Bruuns T, Lee S, Taylor J. Amplification and direct sequencing of fungal ribosomal DNA genes for phylogenetics. In: Innis MA, Gelfand DH, Sninsky JJ, White TJ, editors. PCR Protocols: A Guide to Methods and Applications. San Diego, California: Academic Press; 1990. p. 315-22.

4. Kearse M, Moir R, Wilson A, Stones-Havas S, Cheung M, Sturrock S, et al. Geneious Basic: An integrated and extendable desktop software platform for the organization and analysis of sequence data. Bioinformatics. 2012; 28:1647-9.
