# Supplementary material for: Quantitative real-time PCR as a promising tool for the detection and quantification of leaf-associated fungal species – A proof-of-concept using Alatospora pulchella
Source: PLoS One. 2017 Apr 6;12(4):e0174634. doi: 10.1371/journal.pone.0174634 (PMC5383034; doi:10.1371/journal.pone.0174634)
Supplement: S2 File — (DOCX) [file pone.0174634.s002.docx]

**S2.** Assay validation for environmental samples

To validate the specificity of the established assay for environmental samples (i.e., leaf-associated microbial communities), leaf-samples from a previous study [1] that had been stored at -20°C were used. In this earlier study, leaf material was microbially colonized in the laboratory both under control conditions (i.e., non-exposed) and under exposure to a fungicide using near-natural microbial communities obtained from an uncontaminated reference stream site. The morphological identification of conidia revealed the highest diversity of aquatic hyphomycetes (up to 14 species) for the control (C) leaf material. DNA was extracted from leaf discs (diameter = 1.6 cm) of the C treatment during the present study following the method described in detail in S1. These extracts were then used during qPCR runs applying different scenarios (Table A): (i) pure leaf extracts (C1, C2, and C3), (ii) leaf extracts mixed with pure *A. pulchella* extract (C1/C2/C3+Alpu), and (iii) leaf extracts mixed with diluted *A. pulchella* extract (dilutions of 4^-2^ to 4^-8^) to mimic environmental samples with different copy numbers of the target species present including one dilution (i.e., 4^-8^; 9.4 fg template DNA in PCR reactions) below the LoD of 9.9 fg DNA. Thereby, the qPCR-protocol was slightly adapted by reducing the volume of added ddH_2_O for set-ups (ii) and (iii) to equalize the added volumes of the leaf extracts and *A. pulchella* extract/dilutions (i.e., 5 µL). Results of the qPCR runs are shown in Table A.

**Table A** qPCR scenarios, amount of template DNA (ng), and Ct values for qPCR runs using mixtures of leaf extracts and *A. pulchella* extract/dilutions to mimic environmental samples.

| Scenario | Template DNA in PCR (ng) | Ct value |
| --- | --- | --- |
| C1 / C2 / C3 | - | 25.70 / 40.23 / 38.28 |
| C1+Alpu / C2+Alpu / C3+Alpu | 123.75 | 13.24 / 15.07 / 15.12 |
| C1+Alpu 4^-2^ / C2+Alpu 4^-2^ / C3+Alpu 4^-2^ | 7.75 | 16.95 / 18.87 / 18.56 |
| C1+Alpu 4^-4^ / C2+Alpu 4^-4^ / C3+Alpu 4^-4^ | 2.4 | 21.18 / 23.00 / 22.85 |
| C1+Alpu 4^-6^ / C2+Alpu 4^-6^ / C3+Alpu 4^-6^ | 0.15 | 24.49 / 27.15 / 26.84 |
| C1+Alpu 4^-8^ / C2+Alpu 4^-8^ / C3+Alpu 4^-8^ | 9.4 × 10^-3^ | 26.15 / 30.09 / 29.20 |

References cited in S2

1. Bundschuh M, Zubrod JP, Kosol S, Maltby L, Stang C, Duester L, et al. Fungal composition on leaves explains pollutant-mediated indirect effects on amphipod feeding. Aquat. Toxicol. 2011;104:32-37.
