# Supplementary material for: Quantitative real-time PCR as a promising tool for the detection and quantification of leaf-associated fungal species – A proof-of-concept using Alatospora pulchella
Source: PLoS One. 2017 Apr 6;12(4):e0174634. doi: 10.1371/journal.pone.0174634 (PMC5383034; doi:10.1371/journal.pone.0174634)
Supplement: S3 File — (DOCX) [file pone.0174634.s003.docx]

**S3.** Limit of detection (LoD) and limit of quantification (LoQ)

For qPCR assays, the LoD is defined as the lowest number of PCR forming units (PFU) at which the probability of detection is ≥ 95% (International Organization for Standardization, 2006). In order to determine the LoD, the target DNA PFU was assumed to follow a Poisson distribution in the diluted standards (*Eq.*1; [1]):

$$P\left( x;\lambda\right)=\frac{\lambda^{x}e^{-\lambda}}{x!} (Eq. 1)$$

where *P(x;λ)* is the Poisson probability that *x* PFU are included in a PCR, when the expected number of PFU is *λ*, and *e* is the base of the natural logarithm. Under this assumption, the probability to include at least 1 PFU in a PCR reaction (by definition of PFU yielding in a positive PCR) is ≥ 95% when the expected number of PFU in a PCR reaction is 3 (=LoD; *Eq.* 2; [cf., 2]):

$$P\left( \geq1;3 \right)=1-\left( \frac{3^{0}e^{-3}}{0!} \right) (Eq. 2)$$

The LoQ is defined as the lowest number of PFU in a sample that can be reliably quantified with an acceptable degree of measurement uncertainty [3]. During the present study, the observed measurements were Ct values, which were subsequently converted into PFU. Therefore, the standard deviations (SDs) obtained for Ct values were used as measurement uncertainty. Ct values are based on the exponential amplification of the target copies, and therefore a 50% change in PFU corresponds approximately to a ΔCt of 0.6 (2^0.6^ ≈ 1.5; per PCR cycle, the amount of target copies is doubled). With an accepted uncertainty of ≤ 50% (SD ≤ 0.5), the Ct value of the eighth standard was used to calculate the theoretical LoQ using an exponential function (Eq. 3; Table 2):

$$LoQ \left( PFU \right)={Ct}_{4^{-8}} \times2^{0.6} (Eq. 3)$$

where ${Ct}_{4^{-8}}$ is the Ct value of the eighth standard solution.

References cited in S3

1. Berdal KG, Holst-Jensen A. Roundup Ready^®^ soybean event-specific real-time quantitative PCR assay and estimation of the practical detection and quantification limits in GMO analyses. Eur Food Res Technol. 2001; 213:432-8.

2. Bustin SA, Benes V, Garson JA, Hellemans J, Huggett J, Kubista M, et al. The MIQE Guidelines: Minimum Information for Publication of Quantitative real-time PCR experiments. Clinical Chemistry. 2009; 55:611-22.

3. International Organization for Standardization. ISO 24276; Foodstuffs - Methods of analysis for the detection of genetically modified organisms and derived products - General requirements and definitions. Geneva, Switzerland: International Organization for Standardization; 2006.
