# Supplementary material for: Quantitative real-time PCR as a promising tool for the detection and quantification of leaf-associated fungal species – A proof-of-concept using Alatospora pulchella
Source: PLoS One. 2017 Apr 6;12(4):e0174634. doi: 10.1371/journal.pone.0174634 (PMC5383034; doi:10.1371/journal.pone.0174634)
Supplement: S4 File — (DOCX) [file pone.0174634.s004.docx]

**S4.** Theoretical validation of the qPCR assay’s specificity.

The specificity of the qPCR assay was theoretically validated in two steps. At first, the sequences for the *A. pulchella* strains included in the present study were used to identify the genetically closest species. Therefore, a BLASTn-search at NBCI was conducted and the 100 sequences with the highest query cover were downloaded and aligned using ClustalW [1]. jModelTest 2 [2] was then applied to identify the optimal nucleotide substitution model judged by the Bayesian information criterion. A Kimura 2-parameter model with a gamma distribution of mutations and a proportion of invariant sites (K80+G+I) was found to be the model fitting the data best. The open source software MEGA version 6.06 for Mac [3] was used to generate a Maximum Likelihood tree to visualize relationship patterns. The model was set to K80 and a gamma distributed rate among sites with gamma categories set to 5 was selected. As heuristic model, the nearest-neighbour-interchange was chosen and the initial tree was constructed automatically. Gaps and missing data were treated with the option complete deletion and 1000 bootstrap replicates were generated. The sequences with the closest genetic relationship to *A. pulchella* (Fig. A) were extracted from the dataset. In a second step, further sequences for the ITS-region of morphologically most related species (i.e., all remaining sequences of the genus *Alatospora*) were downloaded from NCBI. Finally, the subset of sequences from genetically as well as morphologically closely related species were aligned together with the sequence motifs for primers and probe to validate its specificity (Fig. B).

**Fig. A** Unrooted tree, based on Maximum Likelihood, for the 100 genetically closest-related sequences (downloaded from NCBI) when compared to the *A. pulchella* strains used during the present study. The red frame indicates sequences used for further specificity analysis of the probe based on genetic similarity. Note that only sequences identified to the species level were used for further specificity analysis. Accession numbers at NCBI are given in parentheses.

|  |
| --- |
| **Fig. B** Sequence motifs of the qPCR primers and probe (highlighted in blue) in the ITS-1 region and sequences of species displaying the closest genetic or morphological relationship to the *A. pulchella* strains used during the present study. Accession numbers at NCBI are given in parentheses. Colours indicate base mismatches at the respective sequence positions. |

References cited in S4

1. Higgins DG, Thompson JD, Gibson TJ. Using CLUSTAL for multiple sequence alignments. In: Russell FD, editor. Methods in Enzymology. Volume 266: Academic Press; 1996. p. 383-402.

2. Darriba D, Taboada GL, Doallo R, Posada D. jModelTest 2: more models, new heuristics and parallel computing. Nat Meth. 2012; 9(8):772.

3. Tamura K, Stecher G, Peterson D, Filipski A, Kumar S. MEGA6: Molecular Evolutionary Genetics Analysis Version 6.0. Molecular Biology and Evolution. 2013; 30(12):2725-9.
