# Supplementary material for: Quantitative real-time PCR as a promising tool for the detection and quantification of leaf-associated fungal species – A proof-of-concept using Alatospora pulchella
Source: PLoS One. 2017 Apr 6;12(4):e0174634. doi: 10.1371/journal.pone.0174634 (PMC5383034; doi:10.1371/journal.pone.0174634)
Supplement: S5 File — (DOCX) [file pone.0174634.s005.docx]

**S5.** Estimated evolutionary divergences among sequences.

To assess genetic distances among the analysed aquatic hyphomycetes, evolutionary divergences between the retrieved sequences were calculated using MEGA 6.06 for Mac [1]. For the target species *A. pulchella*, the lowest genetic divergence – and therefore the closest genetic relationship – was found in species from the same genus (i.e., *A. acuminata* and *A. flagellata;* Table A). Calculations resulted in 0.028 and 0.033 base differences in sites when comparing *A. pulchella* and *A. acuminata* and *A. pulchella* and *A. flagellata*, respectively (Table A).

**Table A.** Number of base differences per site between sequences, involving 15 nucleotide sequences. All positions containing gaps and missing data were eliminated, with a total of 427 positions in the final dataset.

|  | *A.*  *acuminata* | *A.*  *flagellata* | *A.*  *pulchella* | *A.*  *crassa* | *A.*  *filiformis* | *A.*  *atra* | *L.*  *cornuta* | *L.*  *terrestris* | *T.*  *breve* | *T.*  *furcatum* | *T.*  *maxilliforme* | *T. setigerum* | *T. angulum* | *T. chaetocladium* |
| --- | --- | --- | --- | --- | --- | --- | --- | --- | --- | --- | --- | --- | --- | --- |
| *Alatospora acuminata* |  |  |  |  |  |  |  |  |  |  |  |  |  |  |
| *A. flagellata* | 0.023 |  |  |  |  |  |  |  |  |  |  |  |  |  |
| *A. pulchella* | 0.028 | 0.033 |  |  |  |  |  |  |  |  |  |  |  |  |
| *Anguillospora crassa* | 0.164 | 0.164 | 0.177 |  |  |  |  |  |  |  |  |  |  |  |
| *A. filiformis* | 0.176 | 0.176 | 0.178 | 0.180 |  |  |  |  |  |  |  |  |  |  |
| *Articulospora. atra* | 0.157 | 0.155 | 0.162 | 0.148 | 0.166 |  |  |  |  |  |  |  |  |  |
| *Lemmoniera cornuta* | 0.169 | 0.169 | 0.171 | 0.187 | 0.066 | 0.155 |  |  |  |  |  |  |  |  |
| *L. terrestris* | 0.180 | 0.180 | 0.183 | 0.199 | 0.075 | 0.169 | 0.023 |  |  |  |  |  |  |  |
| *Tetracladium breve* | 0.173 | 0.164 | 0.162 | 0.164 | 0.197 | 0.145 | 0.173 | 0.185 |  |  |  |  |  |  |
| *T. furcatum* | 0.183 | 0.173 | 0.171 | 0.169 | 0.194 | 0.155 | 0.178 | 0.180 | 0.028 |  |  |  |  |  |
| *T. maxilliforme* | 0.176 | 0.171 | 0.169 | 0.171 | 0.201 | 0.166 | 0.183 | 0.185 | 0.030 | 0.014 |  |  |  |  |
| *T. setigerum* | 0.178 | 0.169 | 0.166 | 0.169 | 0.194 | 0.159 | 0.176 | 0.178 | 0.0223 | 0.007 | 0.007 |  |  |  |
| *Tricladium angulum* | 0.157 | 0.157 | 0.152 | 0.164 | 0.162 | 0.143 | 0.157 | 0.162 | 0.133 | 0.124 | 0.133 | 0.126 |  |  |
| *T. chaetocladium* | 0.159 | 0.155 | 0.159 | 0.152 | 0.173 | 0.054 | 0.162 | 0.173 | 0.143 | 0.157 | 0.157 | 0.155 | 0.152 |  |
| *T. splendens* | 0.155 | 0.159 | 0.152 | 0.169 | 0.136 | 0.152 | 0.155 | 0.164 | 0.180 | 0.180 | 0.187 | 0.180 | 0.150 | 0.157 |

Reference cited in S5

1. Tamura K, Stecher G, Peterson D, Filipski A, Kumar S. MEGA6: Molecular Evolutionary Genetics Analysis Version 6.0. Mol. Biol. Evol. 2013; 30:2725-9.
